# Mechanistic insights from animal models of neurofibromatosis type 1 cognitive impairment

**DOI:** 10.1242/dmm.049422

**Published:** 2022-08-29

**Authors:** Andrew H. Miller, Mary C. Halloran

**Affiliations:** 1 Department of Integrative Biology, University of Wisconsin-Madison, Madison, WI 53706, USA; 2 Department of Neuroscience, University of Wisconsin-Madison, Madison, WI 53705, USA; 3 Neuroscience Training Program, University of Wisconsin-Madison, Madison, WI 53705, USA

**Keywords:** Neurofibromatosis, Neurofibromin, Ras signaling, cAMP signaling

## Abstract

Neurofibromatosis type 1 (NF1) is an autosomal-dominant neurogenetic disorder caused by mutations in the gene neurofibromin 1 (*NF1*). NF1 predisposes individuals to a variety of symptoms, including peripheral nerve tumors, brain tumors and cognitive dysfunction. Cognitive deficits can negatively impact patient quality of life, especially the social and academic development of children. The neurofibromin protein influences neural circuits via diverse cellular signaling pathways, including through RAS, cAMP and dopamine signaling. Although animal models have been useful in identifying cellular and molecular mechanisms that regulate NF1-dependent behaviors, translating these discoveries into effective treatments has proven difficult. Clinical trials measuring cognitive outcomes in patients with NF1 have mainly targeted RAS signaling but, unfortunately, resulted in limited success. In this Review, we provide an overview of the structure and function of neurofibromin, and evaluate several cellular and molecular mechanisms underlying neurofibromin-dependent cognitive function, which have recently been delineated in animal models. A better understanding of neurofibromin roles in the development and function of the nervous system will be crucial for identifying new therapeutic targets for the various cognitive domains affected by NF1.

## Abstract (precis)

**Summary:** Neurofibromin influences neural circuits through RAS, cAMP and dopamine signaling. Exploring the mechanisms underlying neurofibromin-dependent behaviors in animal models might enable future treatment of the various cognitive deficits that are associated with neurofibromatosis type 1.

## Introduction

Neurofibromatosis type 1 (NF1) is a multi-symptom neurogenetic disorder caused by heterozygous loss-of-function mutations in the neurofibromin 1 (*NF1*) gene, which encodes neurofibromin, a tumor suppressor that inhibits signaling via the small GTPase rat sarcoma virus (RAS) subfamily of proteins. The birth incidence of NF1 is estimated to be ∼1 in 2000-3000 (Crowe et al., 1956; Evans et al., 2010; Huson et al., 1989; Uusitalo et al., 2015). Although inheritance is autosomal dominant, ∼50% of *NF1* mutations are sporadic, occurring without an established family history (Evans et al., 2010; Huson et al., 1989; McKeever et al., 2008; Messiaen et al., 2000; Sergeyev, 1975). Beginning in childhood, the earliest indications of NF1 are often pigment irregularities, including hyperpigmented skin lesions, skinfold freckling and iris hamartomas (Cimino and Gutmann, 2018). Additionally, neurological features are an important clinical concern. Individuals with NF1 are predisposed to the development of peripheral nerve and brain tumors, including neurofibromas and optic pathway gliomas. However, the most-common neurological feature is cognitive dysfunction (Cimino and Gutmann, 2018). Up to 80% of children with NF1 experience cognitive or behavioral deficits that negatively impact their social and academic development (Hyman et al., 2005; Torres Nupan et al., 2017). Various cognitive domains and behaviors may be altered in patients with NF1, including attention, academic skills, social competence, visuospatial skills, executive function, motor function, memory, language and emotional control (Lehtonen et al., 2013; Torres Nupan et al., 2017). Additionally, there are reports of considerable diagnostic overlap between NF1 and both attention-deficit/hyperactivity disorder (ADHD) and autism spectrum disorder (Eijk et al., 2018; Garg et al., 2013; Hyman et al., 2005; Lehtonen et al., 2013; Plasschaert et al., 2015; Torres Nupan et al., 2017). Therefore, improving cognitive function in patients with NF1 is vital to improve health-related quality of life (Krab et al., 2009; Varni et al., 2019).

NF1 patient care is often complicated by variability in both the manifestation and rate of progression of specific symptoms, even within affected individuals from the same family (Cimino and Gutmann, 2018; Riccardi, 1982). Existing treatments for the cognitive deficits associated with NF1 are very limited, with a small number of studies showing that treatment with stimulants potentially improves attention and general cognition in NF1 patients with ADHD-like symptoms (Lidzba et al., 2014; Lion-Francois et al., 2014; Mautner et al., 2002; Torres Nupan et al., 2017). Animal models have been useful in identifying cellular and molecular mechanisms that regulate NF1-dependent behaviors; however, translating these discoveries into effective treatments to improve cognition in patients with NF1 has not yet been successful. Variability in patient symptoms and efficacy of treatments may reflect differences between specific *NF1* mutations, modifier genes, developmental effects, sex differences and/or the multifunctional activity of neurofibromin in neural circuits. Therefore, investigation of neural circuits in a variety of *Nf1-*mutant animals may be necessary to effectively recapitulate the distinct cognitive dysfunctions experienced by subgroups of patients with NF1, and enable the development of effective new therapies to improve cognitive and behavioral functions.

In this Review, we first describe the structure and function of *NF1* and of neurofibromin. Then, we evaluate several proposed cellular and molecular mechanisms underlying neurofibromin-dependent cognitive function. We focus on recent advances in animal models that delineate the role of neurofibromin in RAS, cyclic adenosine monophosphate (cAMP) and dopamine signaling, and in neuronal excitation-inhibition balance. Throughout, we reflect on how these advances can further the development of effective therapies for NF1 patients.

## NF1-associated gene, protein and cellular signaling

Human *NF1* is a large gene of ≥280 kb and 57 constitutive exons, located on chromosome 17q11.2 (Barker et al., 1987; Cawthon et al., 1990; Marchuk et al., 1991; Seizinger et al., 1987; Viskochil et al., 1990; Wallace et al., 1990). Four additional alternatively spliced *NF1* exons generate transcript isoforms with up to 60 or 61 total exons (reviewed by Bergoug et al. 2020), with updated exon nomenclature proposed by Anastasaki et al. (2017). The *NF1* transcript variants generated by alternative splicing differ in their level and pattern of expression (Andersen et al., 1993; Gutman et al., 1993; Nishi et al., 1991; Park et al., 1998; Vandenbroucke et al., 2002). At the time of writing, 2660 *NF1* mutations have been recorded in the Human Gene Mutation Database (HGMD^®^) (Stenson et al., 2020). The large number of unique *NF1* mutations located broadly throughout the gene have complicated correlations between mutation and the manifestation or severity of NF1 symptoms. However, more recently, correlations have emerged between specific mutations and groups of symptoms, including neurofibromas (Pinna et al., 2015; Upadhyaya et al., 2007), Noonan-like features (Rojnueangnit et al., 2015) and the development of optic pathway gliomas (Anastasaki et al., 2019; Xu et al., 2018), and an association between large genomic deletions and increased symptom severity has been reported (Kehrer-Sawatzki et al., 2017).

**Fig. 1.**
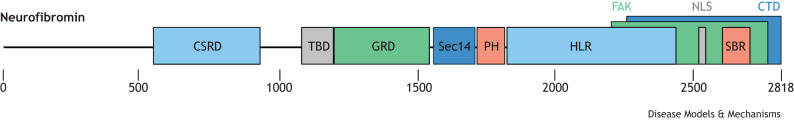
**Schematic of neurofibromin and its predicted structural components.** CSRD, cysteine/serine-rich domain (aa 543-909); CTD, C-terminal domain (aa 2260-2817); FAK, focal adhesion kinase-interacting domain (within aa 2205-2785); GRD, GTPase-activating protein (GAP)-related domain (aa 1198-1530); HLR, HEAT-like repeat region (aa 1825-2428); NLS, nuclear localization signal (aa 2534-2550); PH, pleckstrin homology domain (aa 1716-1816); SBR, syndecan-binding region (aa 2619-2719); Sec14, bipartite lipid-binding module with a Sec14-like domain (aa 1560-1705); TBD, tubulin-binding domain (aa 1095-1197) (Bollag et al., 1993; Bonneau et al., 2004; Bonneau et al., 2009; D'Angelo et al., 2006; Kweh et al., 2009; Scheffzek et al., 1998). aa, amino acids.

**Fig. 2.**
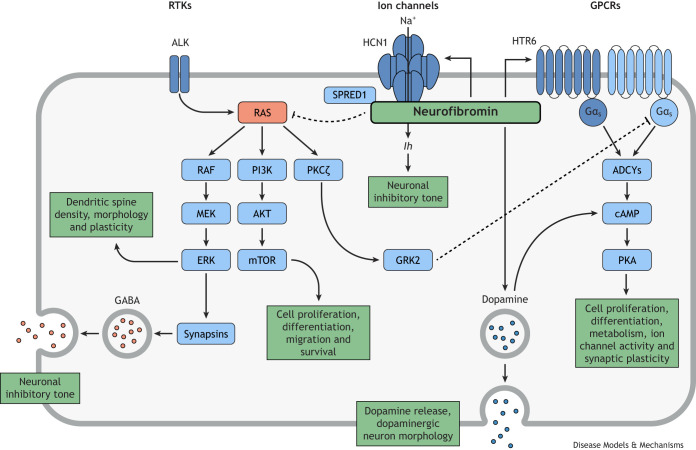
**Neurofibromin-mediated regulation of cellular signaling pathways.** Neurofibromin regulates several signaling pathways, thereby affecting diverse cellular functions. Neurofibromin-mediated inhibition of RAS reduces downstream kinase signaling to regulate neuronal inhibitory tone, dendritic spine plasticity and cellular development. Neurofibromin regulates cAMP signaling in both RAS-dependent and RAS-independent manners, affecting cellular development, metabolism and synaptic plasticity. Additionally, neurofibromin regulates dopamine release and dopaminergic neuron morphology. HTR6, 5-hydroxytryptamine receptor 6; ADCYs, adenylyl cyclases; AKT, AKT serine/threonine kinases; ALK, anaplastic lymphoma kinase; cAMP, cyclic adenosine monophosphate; ERK, extracellular signal-regulated kinases; Gα_s_, heterotrimeric G protein alpha s subunit; GABA, gamma-aminobutyric acid; GRK2, G protein-coupled receptor kinase 2; HCN1, hyperpolarization-activated cyclic nucleotide gated potassium channel 1; MEK, mitogen-activated protein kinase kinases; mTOR, mechanistic target of rapamycin; PI3K, phosphoinositide 3-kinase; PKA, protein kinase A; PKCζ, protein kinase C zeta; RAF, rapidly accelerated fibrosarcoma kinase family proteins; RAS, rat sarcoma virus small GTPase family proteins; SPRED1, sprouty related EVH1 domain-containing 1. Figure adapted from Anastasaki et al. (2022).

The *NF1* gene product neurofibromin is a multifunctional, cytoplasmic protein involved in several cellular signaling pathways. The full-length human *NF1* transcript encodes 2818 amino acids (Anastasaki et al., 2017; Marchuk et al., 1991), and multiple neurofibromin protein domains have been identified (outlined in Fig. 1) (Bollag et al., 1993; Bonneau et al., 2004; Bonneau et al., 2009; D'Angelo et al., 2006; Kweh et al., 2009; Scheffzek et al., 1998). Generally, *NF1* is ubiquitously expressed during development but, in adulthood, expression is most abundant in neurons, oligodendrocytes, non-myelinating Schwann cells and leukocytes (Daston et al., 1992; Daston and Ratner, 1992; Gutmann et al., 1991, 1995a,b; Wallace et al., 1990). Within cells, neurofibromin is primarily cytoplasmic but can be transported to the nucleus (Li et al., 2001; Vandenbroucke et al., 2004). In the cytoplasm, neurofibromin is translocated to the plasma membrane by binding to sprouty-related, EVH1 domain-containing protein 1 (Spred1), which facilitates interaction with RAS (Fig. 2) (Dunzendorfer-Matt et al., 2016; Stowe et al., 2012).

The first insights into a functional role for neurofibromin came from studies of its regulation of RAS signaling. Sequence analysis showed similarities between mammalian *Nf1* and yeast *IRA1* and *IRA2* gene products, which are homologous to mammalian guanosine triphosphate (GTP)ase-activating proteins (GAPs) (Ballester et al., 1990; Buchberg et al., 1990; Tanaka et al., 1990a,b; Xu et al., 1990b). RAS-specific GAPs, like neurofibromin, inhibit RAS signaling by facilitating the hydrolysis of active GTP-bound RAS to inactive guanosine diphosphate (GDP)-bound RAS (Ballester et al., 1990; Martin et al., 1990; Trahey and McCormick, 1987; Xu et al., 1990a). Small GTPase RAS proteins, such as human H-RAS, K-RAS and N-RAS, are controlled like binary switches, which then regulate intracellular signaling. Loss of the inhibitory effects of neurofibromin leads to hyperactivation of downstream RAS signaling, including – but not limited to – the canonical RAS-RAF-ERK signaling pathway (Box 1, Fig. 2) (Basu et al., 1992; Dasgupta et al., 2005; DeClue et al., 1991; Endo et al., 2013; Johannessen et al., 2005). These phosphorylation cascades downstream of RAS regulate cell proliferation, differentiation, migration and survival, and are often targeted in the treatment of cancers, as reviewed by Asati et al. (2016).

**Box 1. Glossary**

**AKT kinases:** serine/threonine-specific protein kinases that have a role in cellular processes, e.g. apoptosis, cell proliferation and transcription.

**Blood-brain barrier:** barrier formed by endothelial cells that protects the brain from toxins and pathogens, and regulates the movement of cells, molecules and ions into and out of the central nervous system.

**Catecholaminergic neuron-targeted vector-assisted spectral tracing:** adeno-associated viral vector-based tool that targets catecholaminergic neurons. The tool is controlled by the tyrosine hydroxylase promoter and provides sparse multi-color labeling for visualization of cell morphology.

**Contextual fear conditioning:** behavioral test to quantify fear learning, in which an animal associates a neutral environment with an aversive stimulus. After repeated exposure to the aversive stimulus, reintroduction to the neutral environment elicits escape or defensive behaviors.

**Cued fear conditioning:** behavioral test to quantify fear learning, in which an animal associates a neutral stimulus with an aversive stimulus. After repeated pairings of neutral and aversive stimuli, exposure to the neutral stimulus alone elicits escape or defensive behaviors.

**Dendritic spine plasticity:** small postsynaptic membranous protrusions, i.e. dendritic spines, are found along neuronal dendrites. Spine shape, volume and density is highly dynamic, and shaped during development and through neural activity.

**Extracellular signal-regulated kinases (ERKs):** see mitogen-activated protein kinases (MAPKs).

**GABAergic tone:** level of neuronal inhibition provided by signaling through gamma-aminobutyric acid (GABA) receptors.

**Glial fibrillary acidic protein (GFAP):** intermediate filament protein used as a marker for astrocytes in the brain. During development, GFAP is detected in radial glial cells – progenitor cells for neurons, astrocytes and oligodendrocytes.

**Habituation:** simple form of non-associative learning that is observed as a progressive decline in responsiveness to repeated and non-harmful stimuli.

**Hippocampal CA1 pyramidal neurons:** the hippocampus is a brain region located in the medial temporal lobe that is crucial for memory, navigation, and cognition. The hippocampal subfield CA1 has densely packed pyramidal neurons that are named according to the shape of their cell bodies. The CA1 region receives information both directly and indirectly from the entorhinal cortex, and constitutes a main output pathway of the hippocampus.

**Long-term potentiation (LTP):** an activity-driven, long-lasting strengthening of synaptic transmission between neurons. LTP contributes to synaptic plasticity during learning and memory.

**Looming visual stimulus assay:** a visual test designed for rodents to mimic an approaching aerial predator. An expanding dark disc is displayed above the rodent that elicits escape or freezing behaviors.

**Medial prefrontal cortex (mPFC):** a subregion of the cerebral cortex that regulates cognitive function, including attention, inhibitory control and memory.

**MEKs:** see mitogen-activated protein kinase kinases (MAP2Ks).

**Mitogen-activated protein kinases (MAPKs):** family of serine/threonine that regulate several cellular functions, e.g. gene expression, proliferation, differentiation. Also known as ERKs.

**Mitogen-activated protein kinase kinases (MAP2Ks)**: Family of dual-specificity kinases that phosphorylate MAPK proteins. Also known as MEKs.

**Morris water maze:** swimming navigation task, in which rodents are trained to use spatial cues to find a hidden platform.

**Olfactory associative learning task:** behavioral task used to quantify learning, in which an animal associates a neutral odor with an aversive stimulus. After repeated pairings of the neutral odor and aversive stimulus, the animal's preference for the paired odor or a different neutral odor is quantified.

**p21 protein-activated kinase (Pak1):** serine/threonine kinase that activates mitogen-activated protein kinases (MAPKs, also known as ERK proteins) and is downstream of Rho family GTPases.

**Platform-removed probe trials:** Morris water maze trial, in which the hidden platform is removed. Time spent in the quadrant where the platform had previously been located is often used as a measure of memory.

**RAF (rapidly accelerated fibrosarcoma) kinases:** family of serine/threonine kinases comprising ARAF, BRAF and RAF1, functioning in the MAPK signaling pathway.

**RAS (rat sarcoma virus) kinases:** family of GTPases comprising, amongst others, HRAS, KRAS and NRAS. They function as molecular switches that control intracellular signaling networks, such as the MAPK pathway.

**RAS-RAF-MEK-ERK signaling pathway** (also known as MAPK pathway): kinase signaling pathway that relays extracellular signals to regulate cellular function, including growth, proliferation and neuronal signaling.

**Spontaneous dopaminergic transient:** increase in dopamine release measured by genetically encoded fluorescent dopamine sensors that detect extracellular dopamine concentrations with sub-micromolar and sub-second resolution.

**Spontaneous inhibitory postsynaptic current:** spontaneous neurotransmitter release from presynaptic cells that gives rise to miniature ionic currents in postsynaptic cells, which can be measured by patch-clamp electrophysiological techniques. GABAergic and glycinergic neurons produce inhibitory currents that decrease the likelihood of the postsynaptic cell to fire an action potential.

**T-maze discrimination task:** behavioral task to quantify learning and reference memory, in which an animal finds a reward by navigating to the left or right arm of a T-shaped apparatus.

**Working memory:** cognitive ability, in which limited amounts of information are temporarily stored and manipulated in the mind enabling the performance of higher-order executive tasks.

A second intracellular signaling pathway that neurofibromin regulates is the cAMP-dependent protein kinase A (PKA) pathway (Fig. 2). The identification of a conserved *Drosophila NF1* homolog first implicated *NF1* in the regulation of cAMP-PKA signaling (The et al., 1997). Subsequently, results from flies, mice and human induced pluripotent stem cells (iPSCs) have led to suggestions that neurofibromin may regulate cAMP-PKA signaling through RAS-independent (Brown et al., 2010b; Brown et al., 2012; Guo et al., 1997), RAS-dependent (Anastasaki and Gutmann, 2014; Walker et al., 2006), or separate RAS-independent and -dependent pathways (Hannan et al., 2006). These signaling pathways induce synthesis of cAMP following activation of adenylyl cyclases (ADCYs) downstream of guanine nucleotide-binding protein (G protein)-coupled receptors (GPCRs) (Anastasaki and Gutmann, 2014; Guo et al., 1997; Hannan et al., 2006). The cAMP effector, PKA, then phosphorylates protein targets to regulate diverse cellular processes, including cell proliferation, differentiation, metabolism, ion channel activity and synaptic plasticity, as reviewed by Taskén and Aandahl (2004).

Besides regulating RAS and cAMP signaling, neurofibromin is known to associate with an abundance of other proteins. In total, the Biological General Repository for Interaction Datasets (BioGRID) lists 133 unique neurofibromin interactions from 55 publications (Oughtred et al., 2021). Notably, these include tubulin (Bollag et al., 1993; Gregory et al., 1993), keratins (Carnes et al., 2019; Koivunen et al., 2000; Malminen et al., 2002), syndecans (Hsueh et al., 2001), kinesin-1 (Hakimi et al., 2002), caveolin-1 (Boyanapalli et al., 2006), β-amyloid precursor protein (De Schepper et al., 2006), the hyperpolarization-activated cyclic nucleotide-gated channel 1 (HCN1) (Omrani et al., 2015) and the serotonin receptor 5-hydroxytryptamine receptor 6 (HTR6) (Deraredj Nadim et al., 2016). Further identification and characterization of neurofibromin protein interactions will be an important step towards a better understanding of its diverse cellular functions.

## Animal models to investigate the role of neurofibromin in cognition and behavior

*Nf1*-mutant animals have been used to study the function of neurofibromin in several species, including mice, flies, zebrafish and minipigs, thereby aiding the identification of a broad range of behaviors that may be regulated by neurofibromin, as described below. Traditionally, experiments using *Nf1-*mutant animals have focused on monitoring learning and memory (Table 1). However, as children with NF1 also display atypical attention and social behaviors, additional tasks have been studied in animal models to recapitulate the diverse cognitive deficits observed in patients (Table 2). Recent progress in this area includes measuring hyperactive grooming (King et al., 2016; King et al., 2020) and aberrant courtship (Moscato et al., 2020) in *Nf1-*mutant flies, as well as assessing hyperactivity and impulsivity (Lukkes et al., 2020), maternal separation-induced vocalizations (Maloney et al., 2018) and long-term social novelty preference (Molosh et al., 2014; Petrella et al., 2016; Shih et al., 2020) in *Nf1-*mutant mice.

**Table 1.**
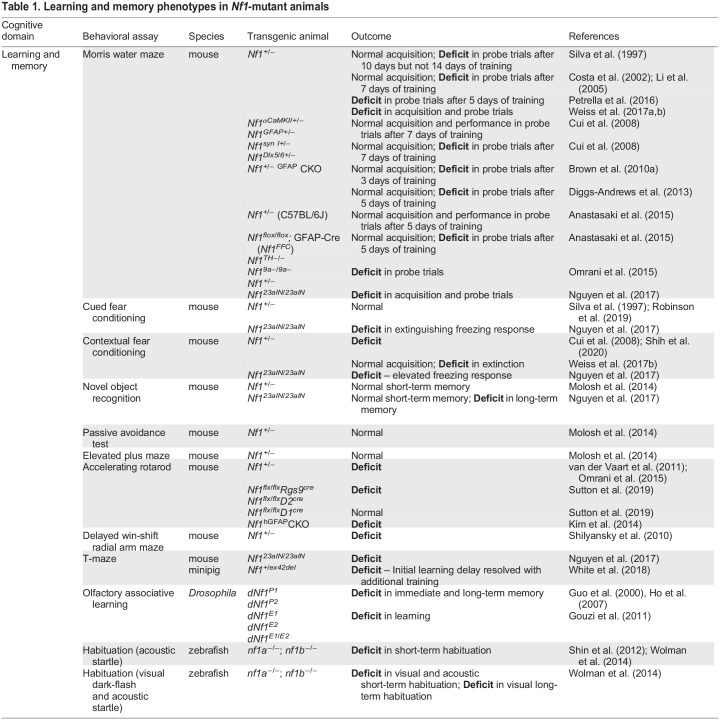
Learning and memory phenotypes in *Nf1*-mutant animals

**Table 2.**
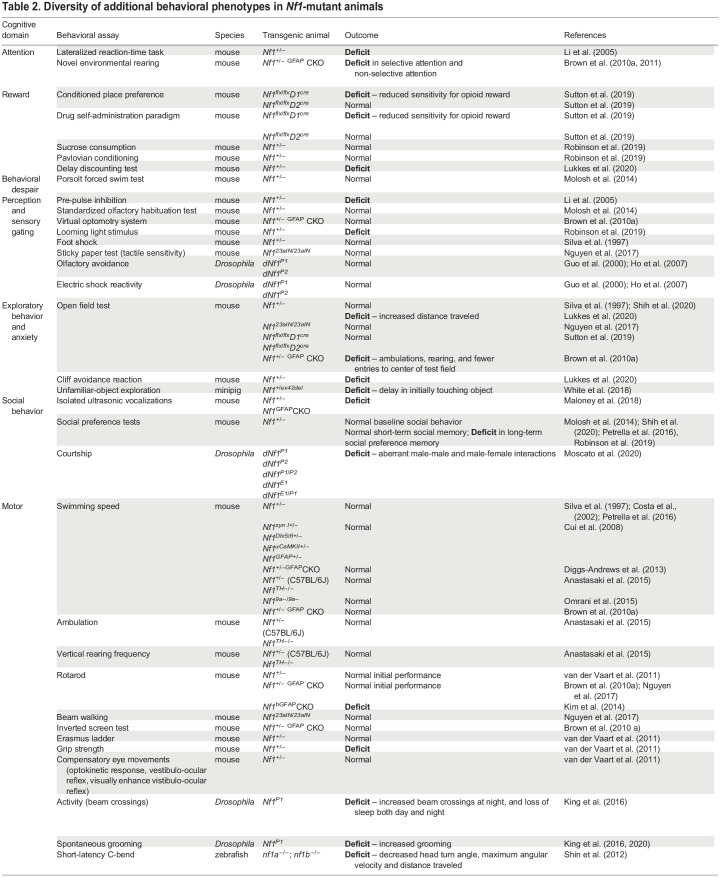
Diversity of additional behavioral phenotypes in *Nf1*-mutant animals

We now review a selection of studies in animal models of NF1 that identified cellular and molecular mechanisms – including RAS, cAMP and dopamine signaling, and neuronal excitation-inhibition balance – which may underlie neurofibromin-dependent behaviors. We also highlight multiple points of overlap between these mechanisms. Advances in exploring the mechanisms that underlie neurofibromin-dependent cognition and behavior in animal models could lead to new therapeutic targets for improving cognitive function in patients with NF1.

### RAS signaling

A series of pioneering studies identified neurofibromin-dependent RAS signaling as able to regulate learning and memory (Costa et al., 2002; Cui et al., 2008; Li et al., 2005; Silva et al., 1997). Mice with heterozygous null mutations in *Nf1* (*Nf1*^+/−^) were used to model the human autosomal dominant condition of NF1 and identified as displaying decreased performance in the Morris water maze (Box 1) (Silva et al., 1997). This deficit in platform-removed probe trials (Box 1) could be rescued with extended training (Silva et al., 1997) and might be representative of a deficit in visuospatial learning and/or memory. More-recent work has shown that the severity of this deficit can be relatively modest, depending on the genetic background and the specific conditional mutant lines or Cre drivers used to reduce levels of neurofibromin (Anastasaki et al., 2015; Diggs-Andrews et al., 2013). Nevertheless, manipulations that decrease RAS-RAF-MEK-ERK signaling (Box 1) improve the deficits of visuospatial learning and/or memory in *Nf1*^+/−^ mice, when these deficits are detectable on a specific F1 hybrid genetic background (Guilding et al., 2007; Li et al., 2005). Further evidence for RAS-dependent roles in learning came from a study showing that *Nf1*^+/−^ mice have defects in hippocampal long-term potentiation (LTP; Box 1), which were reversed by treatment with a RAS inhibitor (Costa et al., 2002)

In pursuit of new cognitive therapies for NF1 patients, one study found that the HMG CoA reductase inhibitor lovastatin that inhibits RAS activity, can decrease RAS activity in the brain and rescue spatial learning and attention impairments in *Nf1^+/−^* mice (Li et al., 2005). Subsequently, several clinical trials assessed the use of lovastatin or the chemically similar simvastatin on cognitive outcomes in patients with NF1 (Bearden et al., 2016; Krab et al., 2008; Mainberger et al., 2013; Payne et al., 2016; Stivaros et al., 2018; van der Vaart et al., 2013). Disappointingly, results have been varied, with limited treatment effects on cognitive function. Causes for the lack of robust therapeutic effects remain unclear. Potential concerns include the efficacy of statins to inhibit RAS activity, the timing of treatment with respect to human development, and the ability of neurofibromin to regulate signaling pathways apart from the RAS signaling pathway. Although lipophilic statins are thought to readily cross the blood-brain barrier (Box 1) (Botti et al., 1991; Saheki et al., 1994), it might be that other RAS-RAF-MEK-ERK signaling inhibitors are better suited to treat NF1-associated cognitive deficits in human patients.

Several studies have tested the ability of inhibitors of the downstream RAS effector MEK to improve behavioral deficits in *Nf1*-mutant animals. For example, in mice with conditional *Nf1* knockout within glial fibrillary acidic protein (GFAP; Box 1)-expressing cells, transient MEK inhibition during a neonatal window prevented defects in cerebellar development and improved long-term motor performance (Kim et al., 2014). In another study, acute MEK inhibition improved memory recall but not short-term learning of *nf1*-deficient larval zebrafish in a visual habituation (Box 1) paradigm (Wolman et al., 2014). Additionally, the feasibility of pharmacological MEK inhibition has been tested in heterozygous *NF1*-mutant minipigs, which are thought to better recapitulate human physiology and the diverse symptoms observed in patients with NF1 (Isakson et al., 2018). In this minipig model for NF1, comprising a recurrent nonsense mutation that mimics one found in NF1 patients, MEK inhibition reduced phorbol-myristate acid (PMA)-stimulated ERK phosphorylation in peripheral blood mononuclear cells (Isakson et al., 2018). In a second heterozygous *NF1*-mutant model, minipigs exhibited learning deficits during the early acquisition of a T-maze discrimination task (Box 1) (White et al., 2018). Therefore, minipigs could prove useful for future NF1 cognitive and behavioral research. Excitingly, work modulating the RAS-RAF-MEK-ERK signaling pathway has led to the recent development of a successful therapeutic for inoperable, symptomatic plexiform neurofibromas in children with NF1. The first-of-its-kind treatment is the U.S. Food and Drug Administration-approved MEK inhibitor selumetinib that shrinks tumor volume and reduces neurofibroma-related pain (Casey et al., 2021; Gross et al., 2020). Although, cognitive outcomes have not been assessed in clinical trials of selumetinib, a cognitive study conducted across multiple ongoing MEK inhibitor trials in NF1 patient populations found potential benefits to some measures of memory (Walsh et al., 2021), indicating that MEK inhibitors might prove more effective than statins. In summary, MEK inhibition improves some neurofibromin-dependent behavioral deficits in animal models and is currently being evaluated for its ability to improve cognition in patients with NF1. However, questions remain, including which specific cognitive behaviors are regulated by RAS signaling and how neuronal activity is modulated to regulate these behaviors.

**Fig. 3:**
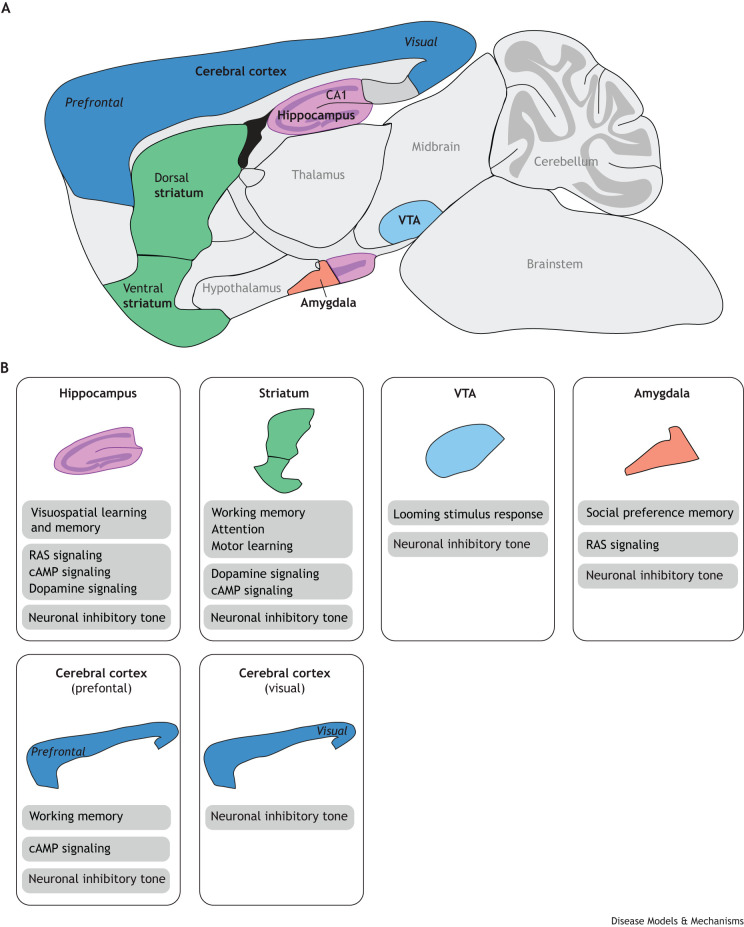
**Brain structures associated with neurofibromin-dependent behaviors and cellular functions.** (A) Schematic showing the lateral view of the mouse brain. Structures indicated in bold are associated with neurofibromin-regulated functions. (B) Neurofibromin-dependent behaviors, cellular signaling pathways and neuronal mechanisms and their associated brain regions. VTA, ventral tegmental area. Adapted from http://www.gensat.org/imagenavigator.jsp?imageID=4759.

Studies suggest that neurofibromin can regulate learning, memory and social behavior through modulation of RAS-dependent inhibitory synaptic transmission. Costa et al. (2002) showed that *Nf1*^+/−^ mice have increased gamma-aminobutyric acid (GABA)-mediated inhibition that can be reversed by inhibition of RAS. A later study by Cui et al. (2008) showed that loss of neurofibromin is associated with increased ERK-dependent phosphorylation of synapsin I in the hippocampus following a contextual fear conditioning paradigm (Fig. 3). Since synapsins regulate vesicle availability for exocytosis of neurotransmitters (Chi et al., 2003), Cui and colleagues hypothesized that synapsin I activity mediated by neurofibromin via RAS-RAF-MEK-ERK signaling regulates the release of GABA (Fig. 2). Consistent with this idea, *Nf1*^+/−^ mice showed increased GABA release in the hippocampus, which was restored following the pharmacological inhibition of ERK signaling (Fig. 3), and visuospatial learning and/or memory deficits were rescued by inhibition of the GABA_A_ receptor (Cui et al., 2008). In addition to its role in the hippocampus, neurofibromin has been shown to regulate activity-dependent GABA release in prefrontal and striatal inhibitory networks (Fig. 3) (Shilyansky et al., 2010), as neuronal inhibition-dependent deficits of working memory (Box 1) in *Nf1*^+/−^ mice were also rescued by inhibition of the GABA_A_ receptor. The behavioral deficit was associated with increased frequency of spontaneous inhibitory postsynaptic currents (Box 1) in the medial prefrontal cortex (mPFC; Box 1) and striatum of *Nf1*^+/−^ mice, which were restored by MEK inhibition (Fig. 3) (Shilyansky et al., 2010). Subsequently, Molosh et al. (2014) hypothesized that reducing hyperactive ERK signaling would rescue altered neuronal inhibition and social behavioral deficits in *Nf1*^+/−^ mice. Genetic deletion of p21 protein-activated kinase (Pak1; Box 1) restored normal frequency of spontaneous inhibitory postsynaptic currents and LTP in the amygdala, as well as long-term social preference memory in *Nf1*^+/−^ mice (Fig. 3) (Molosh et al., 2014). Collectively, these studies provide evidence for RAS signaling-dependent neurofibromin regulation of multiple inhibitory circuits that control learning, memory and social behaviors (Fig. 3).

Another way that neurofibromin potentially regulates learning and memory is by modulating RAS-dependent dendritic spine plasticity (Box 1). Sustained activation of RAS following knockdown of neurofibromin in rat hippocampal CA1 pyramidal neurons (Box 1) impairs dendritic spine structural plasticity and leads to loss of spines (Oliveira and Yasuda, 2014). In this study, the authors used a FRET/FLIM RAS activation sensor to show that neurofibromin contributes to ∼90% of RAS inactivation in dendritic spines and is required for rapid inactivation of RAS. Overexpression of the RAS-GAP domain of neurofibromin rescued the dendritic spine phenotypes, which is consistent with the defect being RAS dependent (Oliveira and Yasuda, 2014). These findings corroborate other accounts of impaired dendritic spine formation following loss or knockdown of neurofibromin (Lin et al., 2007; Wang et al., 2011; Shih et al., 2020). However, the last three studies identified different neurofibromin-dependent signaling pathways for the regulation of spine formation, which may or may not be RAS-dependent.

Interestingly, Nguyen et al. (2017) found no obvious effects on the density or morphology of dendritic spines in cultured cerebellar neurons from a novel *Nf1*-mutant mouse. In this model (*Nf1^23aIN/23aIN^*), splice signals surrounding exon 30alt31 (also known as 23a) were changed to increase exon inclusion. The inclusion of exon 30alt31 (23a) inserts 21 amino acids within the neurofibromin GAP-related domain (GRD), thereby weakening its ability to inactivate RAS in yeast and mammalian cell lines (Andersen et al., 1993; Yunoue et al., 2003). Exon 30alt31 (23a) is predominately included in transcripts expressed in neurons of the peripheral nervous system and in glia cells but is excluded from *Nf1* transcripts in neurons of the central nervous system (Gutmann et al., 1995b). A switch from inclusion to exclusion of exon 30alt31 (23a) in the brain occurs during early embryonic development (Gutmann et al., 1995a; Huynh et al., 1994). It is unclear whether the normal dendritic spine morphology observed in cultured *Nf1^23aIN/23aIN^* mouse cerebellar neurons (Nguyen et al., 2017) is cell-type specific or due to the transgenic approach in which RAS-GAP function is reduced by inclusion of exon 30alt31 (23a) without the alteration of other neurofibromin functional domains. Behaviorally, *Nf1^23aIN/23aIN^* mice display deficits in spatial memory and contextual and cued fear conditioning (Box 1) (Nguyen et al., 2017). Therefore, this work demonstrates alternative splicing to be a key modulator of neurofibromin RAS-GAP activity for the regulation of learning and memory in the mouse brain. Future studies are needed to clarify whether dendritic spine plasticity is altered in those brain regions that regulate learning and memory (Fig. 3).

Another protein associated with neurofibromin and the RAS-RAF-MEK-ERK signaling cascade is the anaplastic lymphoma tyrosine kinase (ALK) receptor. Gouzi et al. (2011) showed that *Drosophila* Alk regulates ERK and that attenuation of Alk rescues olfactory learning and memory in homozygous *Nf1-*mutant flies (Gouzi et al., 2011). Later, an association between ALK and NF1 was explored in mice. Genetic and pharmacological inhibition of ALK in heterozygous *Nf1*-mutant mice was shown to rescue some measures of learning and memory in both the Morris water maze and a contextual fear conditioning paradigm (Weiss et al., 2017a,b). This work highlights the interactions between the neurofibromin and receptor tyrosine kinase cellular signaling pathways in regulating learning and memory (Fig. 2).

Evidence from work using *Nf1*-mutant animals shows that neurofibromin modulates RAS signaling in multiple neural circuits to regulate behavior (Fig. 3). However, neurofibromin is also known to regulate cellular signaling independent of RAS. Therefore, leveraging *Nf1* animal model systems to study additional signaling pathways may aid in identifying new therapeutics to improve cognition in patients with NF1.

### cAMP signaling

Around the same time *Nf1-*mutant mice were used to study the role of RAS signaling in learning and memory (Costa et al., 2002; Cui et al., 2008; Li et al., 2005; Silva et al., 1997), Zhong and colleagues used *Drosophila* to show that neurofibromin also regulates cAMP signaling (Guo et al., 1997; Guo et al., 2000; Hannan et al., 2006; Ho et al., 2007). They first showed that *Drosophila* Nf1 regulates cAMP through the rutabaga (rut)-encoded adenylyl cyclase (Guo et al., 1997). Because the *Drosophila* rut mutants displayed learning and short-term memory deficits, the group tested *Nf1-*mutant flies in an olfactory associative learning task (Box 1). *Nf1-*mutants displayed a learning deficit that was rescued by expressing a constitutively active catalytic subunit of cAMP-dependent PKA (Guo et al., 2000). In addition to the learning deficit, a long-term memory deficit was observed in *Nf1-*mutant flies (Ho et al., 2007). Fragments of human neurofibromin were expressed in *Nf1-*mutant flies to explore the effect of distinct protein domains on learning and memory. Learning was rescued by expression of a C-terminal fragment that had previously been shown to be required for G protein-dependent activation of ADCYs (Hannan et al., 2006; Ho et al., 2007). However, long-term memory was only rescued by fragments comprising the GRD, which is known to regulate RAS activity (Ho et al., 2007). The hypothesis that neurofibromin-dependent cAMP signaling is required for learning whereas RAS signaling is required for memory is consistent with results from pharmacological treatment of *nf1*-deficient larval zebrafish during visual habituation assays (Wolman et al., 2014). The authors found that enhancement of cAMP signaling rescued short-term habituation learning but not memory, whereas inhibition of canonical RAS effector pathways rescued memory but not learning. Clearly, increases in cAMP-PKA signaling improves learning behaviors in multiple *Nf1-*mutant animals. However, questions regarding the underlying mechanism remain, including whether neurofibromin-mediated regulation of cAMP signaling is RAS-dependent.

In support of RAS-dependent neurofibromin regulation of cAMP signaling, Anastasaki and Gutmann (2014) revealed a novel mechanism, in which RAS modulates cAMP signaling in human and mouse neurons via atypical protein kinase C zeta (PRKCZ, hereafter referred to as PKCζ). In *Nf1*^+/−^ mouse hippocampal neurons, the proportion of heterotrimeric G protein subunit G alpha (Gα_s_) in its active GTP-bound state – which activates ADCY to produce cAMP (Fig. 2) – was decreased by almost 50%. Pharmacological and genetic reduction of RAS rescued the decreased Gα_s_ activity, cAMP levels and an axonal length defect. Surprisingly, no differences in the downstream RAS effectors AKT, ERK or c-Jun N-terminal kinase (JNK) activation were identified in either *Nf1*^+/−^ mouse hippocampal neurons or NF1 patient iPSC-derived neural progenitor cells (hNF1-NPCs). There was, however, an increase in atypical PKCζ phosphorylation that could be restored with pharmacological or genetic reduction of RAS. In *Nf1*^+/−^ mouse neurons and hNF1-NPCs, they found increased G protein-coupled receptor kinase 2 (GRK2) expression and phosphorylation, which can be activated by PKCs. Inhibition of GRK2 also rescued the decreased Gα_s_ activity and cAMP levels (Fig. 2), and the abnormal axon length in *Nf1*^+/−^ mouse neurons. Therefore, in certain mouse and human neurons, reduced neurofibromin results in overactive RAS signaling that blocks Gα_s_ activation-mediated production of cAMP via a PKCζ-GRK2 pathway, which is separate from the canonical RAF-MEK-ERK or PI3K-AKT-mTOR RAS effector pathways (Fig. 2). Importantly, this noncanonical PKCζ-GRK2 pathway may explain previous findings that excluded RAS pathway hyperactivation as the reason for *Nf1*-mutant phenotypes, because those studies only monitored the canonical RAS effector pathways (Anastasaki and Gutmann, 2014; Brown et al., 2010b; Wolman et al., 2014).

Neurofibromin also regulates cAMP through a separate, RAS-independent, Gα_s_-coupled GPCR-mediated pathway. Deraredj Nadim et al. (2016) recognized an interaction between neurofibromin and the Gα_s_-coupled GPCR HTR6 that regulates agonist-independent activation of Gα_s_ to induce cAMP production (Fig. 2). They showed that HTR6 coimmunoprecipitated with neurofibromin in protein extracts from mouse striatum and that this interaction is dependent on the neurofibromin pleckstrin homology domain. A cell-permeable interfering peptide that disrupts the interaction between neurofibromin and HTR6 strongly reduces basal cAMP levels in human embryonic kidney (HEK)-293 cells, but cAMP production is unaltered by the RAS inhibitor FTI277. These results indicate that cAMP regulation mediated by the interaction between neurofibromin and HTR6 is RAS independent. Furthermore, synthesis of cAMP and phosphorylation of the cAMP-responsive element-binding protein (CREB) are also reduced in extracts of prefrontal cortex tissue obtained from *Nf1*^+/−^ mice, consistent with a role for neurofibromin-dependent modulation of HTR6 Gα_s_ activity in the brain (Fig. 3). Overall, animal model studies have demonstrated that neurofibromin has key functions in regulating cAMP activity. To our knowledge, cAMP or PKA have not yet been directly targeted in clinical trials for patients with NF1 but, due to the effects of cAMP disruption in flies and zebrafish, this therapeutic avenue should be explored further.

### Dopamine signaling

Dopamine signaling has also been linked to neurofibromin-dependent cognitive function. Children with NF1 that experience attention deficits have, historically, been prescribed stimulants, such as methylphenidate, which increase extracellular dopamine in the brain (Volkow et al., 2001). However, a mechanistic relationship between neurofibromin and dopamine signaling was unknown until a series of studies utilizing a novel *Nf1*-mutant mouse comprising one mutant *Nf1* allele in all somatic cells and Cre-driven homozygous *Nf1* inactivation in GFAP-expressing cells – termed *Nf1^+/−^* ^GFAP^ conditional knockout (CKO) or Nf1 optic glioma (OPG) mice (Brown et al., 2010a, 2011; Diggs-Andrews et al., 2013). These mice have defects in dopaminergic projections to the striatum, and defects in behavioral responses to novel objects and environmental stimuli – considered to be measures of attention in rodents (Fig. 3) (Brown et al., 2010a). Treatment of *Nf1^+/−^*^GFAP^ CKO mice with methylphenidate or the dopamine precursor l-DOPA rescues the behavioral responses. As *Nf1^+/−^*^GFAP^ CKO mice have reduced expression or phosphorylation of presynaptic dopamine markers but intact expression of postsynaptic dopamine receptors in the striatum, a presynaptic defect in dopamine homeostasis might contribute to their behavioral deficits (Brown et al., 2010a, 2011). Interestingly, pharmacological treatments targeting RAS or cAMP signaling do not rescue the attention deficits of *Nf1^+/−^*^GFAP^ CKO mice (Brown et al., 2011). However, in a separate study of dopaminergic projections to the hippocampus in *Nf1^+/−^*^GFAP^ CKO mice, deficits in visuospatial learning and/or memory have been associated with reduced cAMP levels that could be rescued with l-DOPA treatment (Fig. 3) (Diggs-Andrews et al., 2013). Collectively, these results demonstrate that neurofibromin modulates presynaptic dopaminergic signaling that is important for attention, learning and memory. In a more-recent study, Sutton et al. (2019) found that pharmacological activation of cAMP is sufficient to rescue motor learning deficits in mice that carry a loss of *Nf1* in striatal medium spiny neurons that express the D2R dopamine receptor (*Nf1^flx/flx^D2^cre^* mice) (Fig. 3). This study describes a reduction in baseline cAMP levels and total activatable ADCY content that is mediated by dopamine. Taken together, neurofibromin-mediated regulation of dopaminergic signaling may alter different downstream signaling pathways in neural circuits controlling different behaviors. In the striatum, a cAMP-dependent pathway regulates motor learning behaviors, whereas a cAMP-independent pathway contributes to the regulation of attention (Figs 2 and 3).

Robinson et al. (2019) advanced knowledge of the role of neurofibromin in regulating dopamine neurotransmission by monitoring dopamine in awake, active mice. The authors used a genetically encoded dopamine sensor (Patriarchi et al., 2018) to measure extracellular fluorescent dopamine signals in the ventral striatum of *Nf1*^+/−^ mice (Robinson et al., 2019). At baseline, the frequency of spontaneous dopaminergic transients (Box 1) was lower in *Nf1*^+/−^ mice. Electrophysiological recordings in acute midbrain slices demonstrated that *Nf1*^+/−^ dopaminergic neurons are less excitable, with lower spontaneous firing rates due to increased GABAergic tone (Box 1, Fig. 2). To assess dopaminergic neuron morphology *in vivo*, Robinson et al. used a novel tool to assess catecholaminergic neuron-targeted vector-assisted spectral tracing (Box 1) and found *Nf1^+/−^* dopaminergic neurons to have smaller cell bodies but similar neurite complexity and length. The normal neurite morphology in *Nf1*^+/−^ mice differs from that previously observed in cultured *Nf1^+/−^* ^GFAP^ CKO dopaminergic neurons (Brown et al., 2010a), which might reflect differences in neurofibromin expression levels between the heterozygous and conditional KO transgenic lines (Robinson et al., 2019) or differences between the *in vivo* versus *in vitro* approaches. To monitor dopamine signaling during relevant behaviors, Robinson and colleagues measured responses to unconditioned rewards, reward learning and fear conditioning. Through careful experimental design, they found that deficits in a cued fear-conditioning task resulted from atypical responses to salient visual stimuli and were not indicative of differences in learning. In an additional looming visual stimulus assay (Box 1), *Nf1*^+/−^ mice increased their escapes to an available shelter, which was rescued by optogenetically inhibiting non-dopaminergic, mainly GABAergic, neurons in the ventral tegmental area. These results are consistent with a role for neurofibromin regulation of GABAergic neurons in circuits that contribute to behavioral responses to salient visual stimuli.

Because stimulants that alter dopamine signaling are used to treat attention deficits, they have been targeted in clinical trials for patients with NF1. Methylphenidate was shown in initial clinical trials to improve behaviors and attention deficits in children with NF1*,* based on parent and informant reports, and a computer-based attention task (Lion-Francois et al., 2014; Mautner et al., 2002). Expanding on these trials and the work from *Nf1-*mutant animals, a randomized controlled trial to measure the effect of methylphenidate on cognitive function, including sustained attention and spatial working memory, is ongoing (Pride et al., 2018).

### Neuronal excitation-inhibition balance

Neurofibromin has been linked to the regulation of neuronal excitation-inhibition balance. Ryu et al. (2019) showed that *Nf1* mRNA expression is enriched in inhibitory compared to excitatory neurons in wild-type mouse hippocampus and cortex, suggesting that neurofibromin preferentially regulates neuronal inhibitory circuits (Fig. 3). Goncalves et al. (2017) assessed two components of excitation-inhibition balance: the concentration of neurotransmitters, and the levels of GABA_A_ receptors in the hippocampus, striatum and prefrontal cortex of *Nf1*^+/−^ mice. They found that GABA:glutamate ratios are increased in the striatum and cortex, whereas GABA_A_ receptor expression is increased in the hippocampus. Therefore, loss of neurofibromin might alter both pre and postsynaptic regulation of neuronal inhibitory tone (Goncalves et al., 2017). Furthermore, van Lier et al. (2020) investigated the effects of neurofibromin loss during cortical development. In slices of visual cortex from postnatal day 12 and 28 *Nf1*^+/−^ mice, the group found increased neuronal inhibition, suggesting that neurofibromin has important roles in regulating neuronal excitation-inhibition balance during development (Fig. 3). In addition, conditional mutation of *Nf1* during embryonic stages in the medial ganglionic eminence – which gives rise to GABAergic cortical interneurons – led to defects in these neurons at postnatal stages, demonstrating roles for *Nf1* early in development (Angara et al., 2020). These findings underscore the need to consider patient age when designing therapeutic studies, as treatments aimed at rescuing inhibitory tone might need to begin early in life.

As discussed previously in the ‘RAS signaling’ section, RAS-dependent mechanisms for increased inhibition were identified in hippocampal, prefrontal and striatal circuits associated with learning and/or memory (Cui et al., 2008; Shilyansky et al., 2010) and in amygdala-mediated social preference memory (Fig. 3) (Molosh et al., 2014). However, not all the mechanisms by which neurofibromin regulates excitation-inhibition balance are RAS dependent. Omrani et al. (2015) identified the neurofibromin-interacting K^+^/Na^+^ hyperpolarization-activated cyclic nucleotide-gated channel 1 (HCN1), through which neurofibromin regulates neuronal inhibitory tone independent of RAS (Fig. 2). They generated a novel *Nf1* mouse mutant, i.e. *Nf1^9a−/9a−^* mice, comprising limited neuron-specific reduction of neurofibromin by deleting the *Nf1* isoform that contains exon 11alt12 (also known as 9a). These *Nf1^9a−/9a−^* mice are phenotypically similar to *Nf1*^+/−^ mutants, displaying enhanced inhibitory synaptic transmission as well as deficits in hippocampal LTP and visuospatial learning and/or memory. The authors then found the putative interaction between neurofibromin and HCN1, a voltage-gated ion channel that mediates hyperpolarization-activated inward cationic current (*I*_h_). Recordings in *Nf1^9a−/9a−^* inhibitory and excitatory hippocampal neurons revealed a selective reduction of *I*_h_ in GABAergic interneurons, which leads to hyperexcitability in neurons in both the hippocampus and visual cortex (Fig. 3). Importantly, increasing *I*_h_ with the HCN channel agonist lamotrigine rescues the electrophysiological and behavioral deficits in both *Nf1^9a−/9a−^* and *Nf1*^+/−^ mice. Consistent with a RAS-independent mechanism for the regulation of HCN channels, they found no changes in HCN current in interneurons from constitutively active *Hras^G12V^* knock-in mice (Omrani et al., 2015). Collectively, these studies suggest that neurofibromin regulates neuronal inhibitory tone through both RAS-dependent and RAS-independent mechanisms, and that treatments targeting separate mechanisms are enough to restore excitation-inhibition balance and to rescue neurofibromin-dependent behaviors in *Nf1*-mutant animals.

## Conclusions and future directions

Neurofibromin is a multi-functional protein that influences neural circuits through diverse signaling pathways. Although animal models have been useful for identifying cellular and molecular mechanisms of neurofibromin-dependent behaviors, translating these discoveries into effective cognitive treatments for patients has so far been unsuccessful. Previous clinical trials measuring cognitive outcomes in NF1 patients by using statins to inhibit RAS signaling showed limited therapeutic efficacy (Bearden et al., 2016; Krab et al., 2008; Mainberger et al., 2013; Payne et al., 2016; Stivaros et al., 2018; van der Vaart et al., 2013). Causes for the disappointing results remain unclear. One possibility is that the statin treatments did not effectively inhibit RAS signaling and that stronger inhibition of the RAS-RAF-MEK-ERK pathway could lead to cognitive improvements. Therefore, ongoing trials measuring the effects of MEK inhibitors on cognitive outcomes might prove more successful (Walsh et al., 2021). The timing of treatment is another potential concern. The effect loss of neurofibromin has during early development is not addressed by pharmacological treatment beginning in middle or late childhood. Earlier treatment regimens might be necessary to improve some cognitive outcomes. Additionally, neurofibromin might influence other signaling pathways apart from or in combination with RAS to regulate human cognitive function. The multiple interactions between neurofibromin downstream signaling pathways must be taken into consideration when developing therapeutics for patients. It might be necessary to target multiple signaling pathways to successfully treat certain cognitive deficits in patients with NF1. Finally, pharmacological treatments rely on small molecules crossing the blood-brain barrier, a potential issue that should be considered when translating findings from cell and animal studies to human patients. The minipig models, which more closely resemble human physiology, hold promise for future pre-clinical testing of therapeutic approaches.

The efficacy of treatments might also differ among subgroups of NF1 patients with varying severity of dysfunction in separate cognitive domains. These patient subgroups might reflect variations in specific *NF1* mutations, modifier genes and/or the multifunctional activity of neurofibromin in separate neural circuits. Therefore, multiple approaches using a variety of *Nf1-*mutant animals might be necessary to effectively model distinct cognitive dysfunctions. Studies using heterozygous mutant animals that mimic the haploinsufficiency of the human disease, animals genetically engineered to mimic specific patient mutations or conditional homozygous mutant animals, which can reveal novel information about neurofibromin biological functions, are all important avenues of future pursuit.

Behavioral tasks designed to measure learning and memory were frequently used in early studies of *Nf1-*mutant animals. However, in line with current research on the behavior of children with NF1, attention and social behaviors could be increasingly targeted in future animal studies. Measures of behavioral inhibition, impulsivity and hyperactivity could reveal additional mechanisms or circuits through which neurofibromin regulates attention. Regarding social behaviors, several recent studies have identified altered phenotypes in *Nf1-*mutant animals (Table 2). A better understanding of the mechanisms that link loss of neurofibromin to these phenotypes could lead to treatments that improve social function and quality of life in patients with NF1.

The range of cellular functions, neural circuits and behaviors that are disrupted in *Nf1-*mutant animals highlight the multifunctional role of neurofibromin in various cell types and brain regions. As a result, it is unlikely that any single-drug treatment will broadly improve cognitive function in patients with NF1. Although MEK inhibitors have been successful in treating certain tumors associated with NF1 (Casey et al., 2021; Gross et al., 2020), addressing the cognitive aspects of the disease is still an open challenge. Multiple approaches that integrate various animal models and behavioral paradigms are probably necessary to identify treatments for specific cognitive functions in subgroups of patients with NF1. These same principles and approaches are also applicable to investigations of disease mechanisms for other complex diseases that affect cognitive function.
